# Supplementary material for: Assessment of the percentage of full recombinant adeno-associated virus particles in a gene therapy drug using CryoTEM
Source: PLoS One. 2022 Jun 3;17(6):e0269139. doi: 10.1371/journal.pone.0269139 (PMC9165851; doi:10.1371/journal.pone.0269139)
Supplement: S1 Table — (PDF) [file pone.0269139.s001.pdf]

S1 Table

	**Specimen ID**		**Results**					
no.	Sample	repeat	Full particles	Empty particles	Uncertain particles	% F	% E	% U
1	**S2.1**	1	1186	329	5	78	22	0
2		2	1220	304	3	80	20	0
3		3	1234	328	4	79	21	0
4		4	1350	326	4	80	19	0
5		5	1269	325	4	79	20	0
6		6	1293	323	5	80	20	0
7	**S2.5**	1	22	1528	16	1	98	1
8		2	13	1509	13	1	98	1
9		3	19	1474	13	1	98	1
10		4	23	1544	14	1	98	1
11		5	28	1487	19	2	97	1
12		6	23	1524	9	1	98	1

S1 Table. Results from the specificity assessment with the reference full sample **S2.1**, and the reference empty sample **S2.5**. %F; %E and %U represent the percentages of particles classified as full, empty and uncertain, respectively.
