# Supplementary material for: Assessment of the percentage of full recombinant adeno-associated virus particles in a gene therapy drug using CryoTEM
Source: PLoS One. 2022 Jun 3;17(6):e0269139. doi: 10.1371/journal.pone.0269139 (PMC9165851; doi:10.1371/journal.pone.0269139)
Supplement: S2 Table — (PDF) [file pone.0269139.s002.pdf]

S2 Table

	Specimen ID		Results						Statistics		
no.	Sample	Repeat	Full particles	Empty particles	Uncertain particles	% F	% E	% U	mean	stdev	Relative stdev %
1	**S2.1**	1	1186	329	5	78	22	0	79.33	0.75	0.59
2		2	1220	304	3	80	20	0			
3		3	1234	328	4	79	21	0			
4		4	1350	326	4	80	19	0			
5		5	1269	325	4	79	20	0			
6		6	1293	323	5	80	20	0			

S2 Table. Results from the Repeatability assessment on compound **1**. %F; %E and %U represent the percentages of particles classified as full, empty and uncertain, respectively.
