# Supplementary material for: Assessment of the percentage of full recombinant adeno-associated virus particles in a gene therapy drug using CryoTEM
Source: PLoS One. 2022 Jun 3;17(6):e0269139. doi: 10.1371/journal.pone.0269139 (PMC9165851; doi:10.1371/journal.pone.0269139)
Supplement: S3 Table — (PDF) [file pone.0269139.s003.pdf]

S3 Table

Specimen ID			Results			
Occasion/Analyst	Specimen	Theoretical % F	% F repeat 1	% F repeat 2	% F Mean	r^2 value
Occasion 2/ Analyst 1	**S2.1**	79.32	80	79	79.5	0.9974
	**S2.2**	71.30	71	70	70.5	
	**S2.3**	61.28	61	62	61.5	
	**S2.4**	41.24	44	44	44	
Occasion 3/ Analyst 1	**S2.1**	79.32	78	80	79	0.9979
	**S2.2**	71.30	69	71	70	
	**S2.3**	61.28	60	63	61.5	
	**S2.4**	41.24	42	45	43.5	
Occasion 4/ Analyst 1	**S2.1**	79.32	79	81	80	0.9990
	**S2.2**	71.30	73	73	73	
	**S2.3**	61.28	61	63	62	
	**S2.4**	41.24	44	42	43	
Occasion 1/ Analyst 2	**S2.1**	79.32	78	81	79.5	0.9966
	**S2.2**	71.30	72	70	71	
	**S2.3**	61.28	63	64	63.5	
	**S2.4**	41.24	43	42	42.5	
Occasion 2/ Analyst 2	**S2.1**	79.32	81	78	79.5	0.9979
	**S2.2**	71.30	72	71	71.5	
	**S2.3**	61.28	63	63	63	
	**S2.4**	41.24	42	41	41.5	
Occasion 3/ Analyst 2	**S2.1**	79.32	79	79	79	0.9962
	**S2.2**	71.30	71	73	72	
	**S2.3**	61.28	64	64	64	
	**S2.4**	41.24	42	44	43	

S3 Table. Results from the linearity assessment. The “Occasion” represents an imaging session, i.e. a preparation and imaging performed on the same day, using the same equipment, while the “Analyst” represents a microscopist, preparing and imaging the specimen. The theoretical values are calculated as described in Table S5. %F represents the percentage of particles classified as full.
