# Supplementary material for: Assessment of the percentage of full recombinant adeno-associated virus particles in a gene therapy drug using CryoTEM
Source: PLoS One. 2022 Jun 3;17(6):e0269139. doi: 10.1371/journal.pone.0269139 (PMC9165851; doi:10.1371/journal.pone.0269139)
Supplement: S4 Table — (PDF) [file pone.0269139.s004.pdf]

S4 Table

Particle type	VLP (cryoTEM)
Detection method	Ellipse detection
Dark particles	Yes
Maximum particle size (nm)	28
Minimum particle size (nm)	18
Output shape	Circular
Overlapping particles to keep	BestMatchValue
Pre-processing method	Edge Detection
Clear content of detected particle	Yes
Divide large components	No
Edge gap tolerance	0.2
Edge width (nm)	5
Prefer circular ellipses	Yes
Elongatnedness max (≥1)	5
Elongatnedness min (≥1)	1
Polygon smoothing (nm)	0
Post-processing refinement	Yes

S4 Table. VAS detection settings for the image analysis of CryoTEM AAV particles.
