# Supplementary material for: Assessment of the percentage of full recombinant adeno-associated virus particles in a gene therapy drug using CryoTEM
Source: PLoS One. 2022 Jun 3;17(6):e0269139. doi: 10.1371/journal.pone.0269139 (PMC9165851; doi:10.1371/journal.pone.0269139)
Supplement: S1 Fig — (PDF) [file pone.0269139.s006.pdf]

S1 Fig

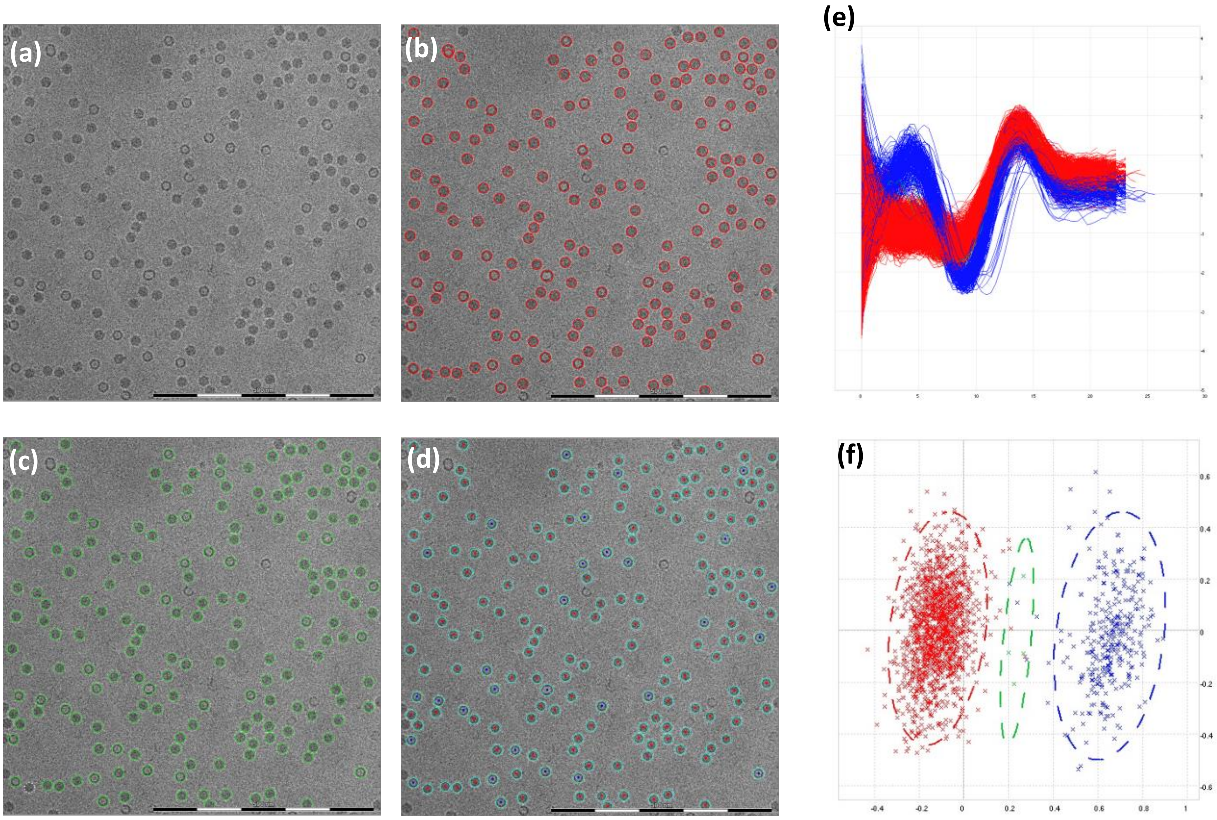

S1 Fig. Intermediate results in the image analysis workflow. (a) Original image. (b) Result after automated detection of particles (overlayed in red). (c) Result after manual correction with the verified particles overlaid in green. (d) Result image after particle classification, with the particles classified as empty marked by a blue dot and particles classified as full marked by a red dot. (e) Radial Density profiles (RDPs), i.e., radial mean intensity curves, for the whole verified particle population from a sample (1680 particles) after classification. (f) Principal Component Analysis of the RDPs, with particles classified as full marked in red, particles classified as empty marked in blue and particles for which the classification was not unambiguous marked in green. The dashed ellipses represent the 99% confidence interval of each particle population, and the scale bars in the images represent 500 nm. Note that in the semi-automatised particle detection, some particles appear to be excluded from the analysis, regardless of their packaging, hence the reliability of the results relies rather on a statistically strong number of detected particles rather than on a faultless detection.
