# Supplementary material for: Assessment of the percentage of full recombinant adeno-associated virus particles in a gene therapy drug using CryoTEM
Source: PLoS One. 2022 Jun 3;17(6):e0269139. doi: 10.1371/journal.pone.0269139 (PMC9165851; doi:10.1371/journal.pone.0269139)
Supplement: S2 Fig — (PDF) [file pone.0269139.s007.pdf]

S2 Fig

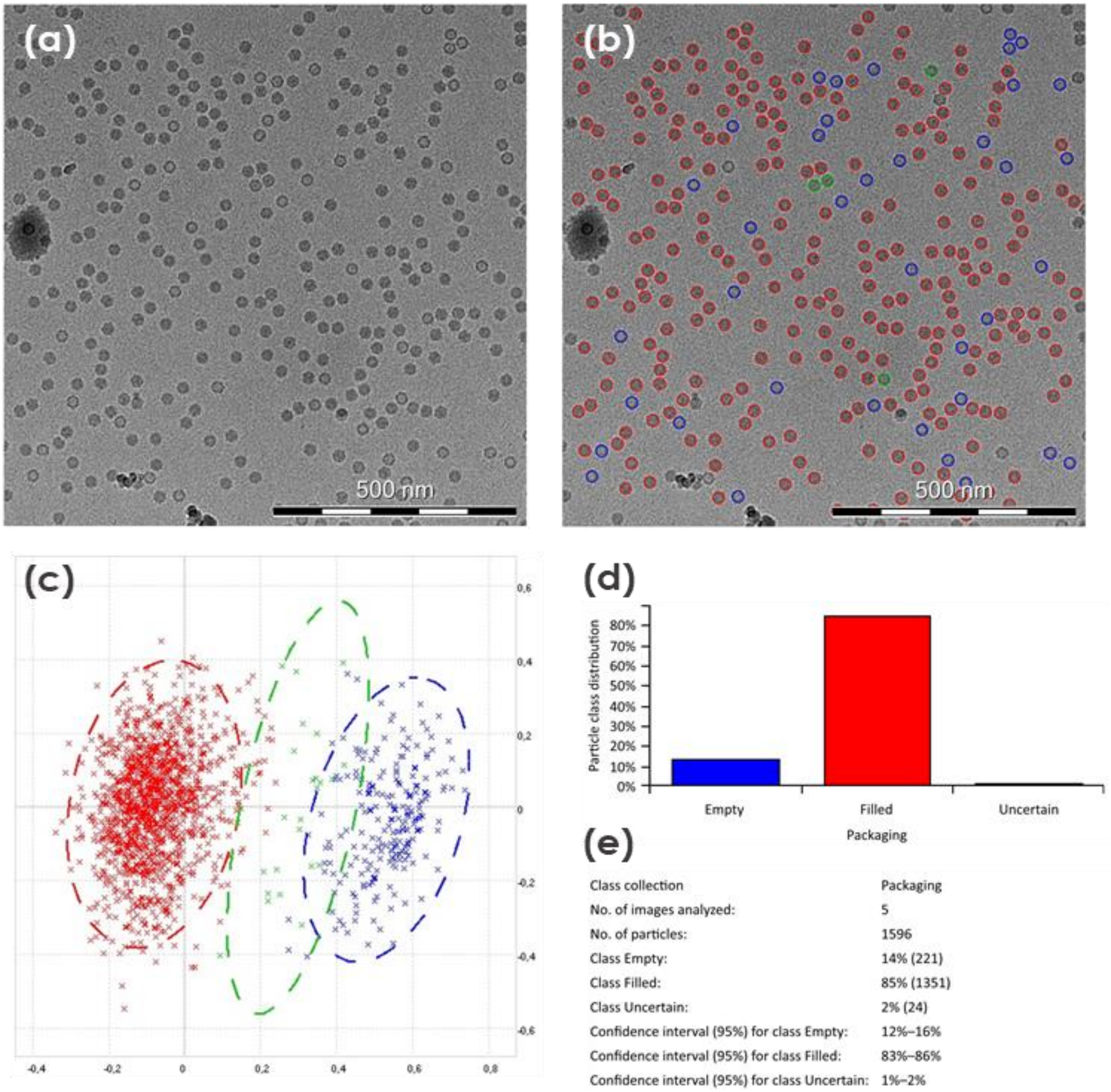

S2 Fig. Packaging assessment on AAV9 particles by CryoTEM. (a) Example original CryoTEM image. (b) Segmented image with detected and classified AAV particles. Particles displaying an inner density with no distinct boundary between the shell and the core are assigned to the class "Full" (Red); particles displaying a distinct outer shell and a minute internal density similar to the background are assigned to the class "Empty" (Blue); particles displaying an intermediate internal

density and detectable outer shell or cannot unambiguously be classified as either "Full" or "Empty" are assigned to the class "Uncertain" (Green). (c) Cluster plot of the detected particles. Dashed ellipses correspond to the 99% confidence interval of the different particle classes. (d) Particle classification diagram. (e) Particle classification statistics.
