# Supplementary material for: Assessment of the percentage of full recombinant adeno-associated virus particles in a gene therapy drug using CryoTEM
Source: PLoS One. 2022 Jun 3;17(6):e0269139. doi: 10.1371/journal.pone.0269139 (PMC9165851; doi:10.1371/journal.pone.0269139)
Supplement: S1 Appendix — (PDF) [file pone.0269139.s008.pdf]

S1 Appendix

The theoretical values used for the linearity assessment were calculated from the whole set of results obtained for the "reference full" samples and the "reference empty" samples in order to give a more accurate value of the theoretical intermediate concentrations by assessing the dilution factor. The experimental mean value for sample **S2.1** "reference full" from 28 individual measurements performed on this sample during the validation study on this sample was calculated to be 79.32%.

	repeat	% F
O1A1	1	78.03
	2	79.90
	3	78.80
	4	80.36
	5	79.41
	6	79.77
O1A2	1	78.18
	2	81.01
O2A1	1	80.14
	2	78.72
O2A2	1	81.12
	2	77.69
O3A1	1	77.52
	2	79.71

	repeat	% F
O3A2	1	78.85
	2	78.55
O4A1	1	78.52
	2	80.65
O5A1	1	79.43
	2	78.63
	3	80.83
	4	78.01
Hold time	1	78.85
	2	79.17
	3	79.92
	4	78.76
	5	80.41
	6	77.82

**Mean**	**79.32**
**Stdev.**	**0.97**

S1 Appendix Table A. Repeats of the reference full specimen **S2.1** used for the calculation of the theoretical values for the linearity assessment. The "O" in "OxAy" corresponds to an Occasion, i.e. an imaging session while "A" represents an individual analyst, preparing and imaging the specimen. The different occasions and analysts were recorded to evaluate the intermediate precision of the method. Note that as not all of the results were used for the

repeatability assessment, the number of samples analysed in each occasion might vary. “Hold time” corresponds to a study in which the specimens were stored 14 days in order to assess their stability to long storage periods.

The experimental mean value for sample **S2.5** “reference empty” from the 6 individual measurements on this sample was calculated to be 1.17%.

	O1A1					
repeat	1	2	3	4	5	6
% F	1	1	1	1	2	1
**Mean:**	**1.17**					
**Stdev.:**	**0.37**					

S1 Appendix Table B. Repeats of the reference full specimen **S2.5** used for the calculation of the theoretical values for the linearity assessment. These experiments were performed on a unique occasion with one microscopist, performing the preparation and analysis.

A calculation of the theoretical value from the dilution factors applied to prepare the samples **S2.2**, **S2.3** and **S2.4** gives the results presented in Table S6. These values were used as reference values for the linearity measurements presented in this study.

Sample	% F
**S2.1**	79.32
**S2.2**	71.30
**S2.3**	61.28
**S2.4**	41.24
**S2.5**	1.17

S1 Appendix Table C. Theoretical values of the percentage of full particles (%F), in the specimen used for the linearity assessment.
