# Supplementary material for: Assessment of the percentage of full recombinant adeno-associated virus particles in a gene therapy drug using CryoTEM
Source: PLoS One. 2022 Jun 3;17(6):e0269139. doi: 10.1371/journal.pone.0269139 (PMC9165851; doi:10.1371/journal.pone.0269139)
Supplement: S2 Appendix — A graphical representation of the standard deviation for each sample size and theoretical full ratio. (PDF) [file pone.0269139.s009.pdf]

S2 Appendix.

In silico study for the determination of a statistically relevant number of detected particles

The minimum number of particles for F/E analysis of AAV particles was assessed using an *in silico* approach. Five randomized datasets containing 5000 data points representing 5000 particles were generated. Each individual datapoint was set to 1 or 0, where 1 represents a full particle and 0 represents an empty particle. The five different data sets contained 1 %, 25 %, 50 %, 75 % or 99 % of "1", representing full particles. From each dataset, 10, 25, 50, 100, 200, 400, 800, 1600 or 3200 particles were randomly sampled and the ratios of full particles were calculated. The process was iterated 100 times and the standard deviation for each sample size and dataset ratio was calculated and plotted (see Figure S3). Sample sizes where the standard deviation is < 2.5 % are acceptable and can be used for determination of the ratio of full particles in a AAV sample, corresponding here to 400 analysed particles. In order to ensure the statistical relevance of the data, a data set of 1500 particles analysed per sample, corresponding to more than 3 times the acceptable number, was used for the data presented in this study.

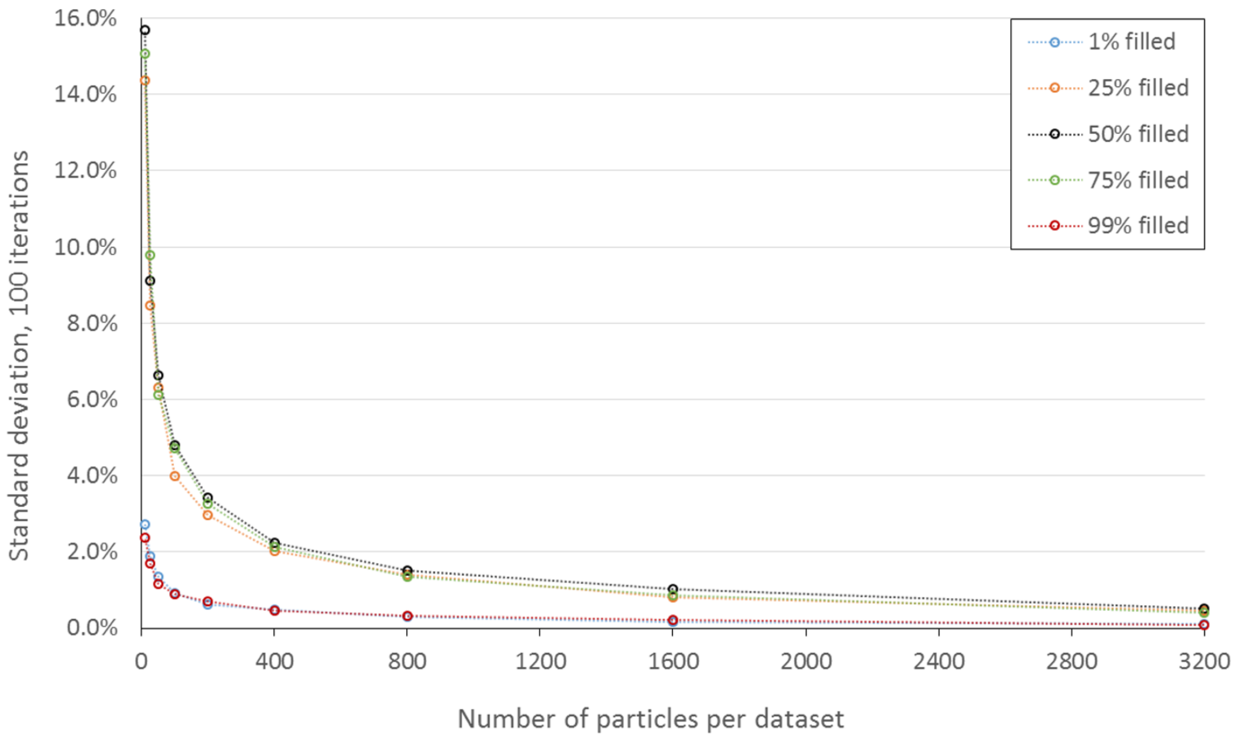

S2 Appendix Fig A. A graphical representation of the standard deviation for each sample size and theoretical full ratio. At a sample size of 400 AAV particles the standard deviation is below 2.5 % for all ratios.

The study shows that when a total of 400 particles are detected, a standard deviation of acceptable < 2.5 % is obtained for all data sets. It is thus concluded that a minimum of 400 particles are sufficient to determine F/E ratio or other binary measurements.
